# Low nucleosome occupancy is encoded around functional human transcription factor binding sites

**DOI:** 10.1186/1471-2164-9-332

**Published:** 2008-07-15

**Authors:** Floris Daenen, Frans van Roy, Pieter J De Bleser

**Affiliations:** 1 Bioinformatics Core, VIB, B-9052 Ghent, Belgium; 2 Molecular Cell Biology Unit, VIB, B-9052 Ghent, Belgium; 3 Department for Molecular Biomedical Research, VIB, B-9052 Ghent, Belgium; 4 Department of Molecular Biology, Ghent University, B-9052 Ghent, Belgium

## Abstract

**Background:**

Transcriptional regulation of genes in eukaryotes is achieved by the interactions of multiple transcription factors with arrays of transcription factor binding sites (TFBSs) on DNA and with each other. Identification of these TFBSs is an essential step in our understanding of gene regulatory networks, but computational prediction of TFBSs with either consensus or commonly used stochastic models such as Position-Specific Scoring Matrices (PSSMs) results in an unacceptably high number of hits consisting of a few true functional binding sites and numerous false non-functional binding sites. This is due to the inability of the models to incorporate higher order properties of sequences including sequences surrounding TFBSs and influencing the positioning of nucleosomes and/or the interactions that might occur between transcription factors.

**Results:**

Significant improvement can be expected through the development of a new framework for the modeling and prediction of TFBSs that considers explicitly these higher order sequence properties. It would be particularly interesting to include in the new modeling framework the information present in the nucleosome positioning sequences (NPSs) surrounding TFBSs, as it can be hypothesized that genomes use this information to encode the formation of stable nucleosomes over non-functional sites, while functional sites have a more open chromatin configuration.

In this report we evaluate the usefulness of the latter feature by comparing the nucleosome occupancy probabilities around experimentally verified human TFBSs with the nucleosome occupancy probabilities around false positive TFBSs and in random sequences.

**Conclusion:**

We present evidence that nucleosome occupancy is remarkably lower around true functional human TFBSs as compared to non-functional human TFBSs, which supports the use of this feature to improve current TFBS prediction approaches in higher eukaryotes.

## Background

Genomic DNA in eukaryotic cells is highly compacted in the nucleus into several levels of chromatin structures that ultimately make up the chromosomes. This compaction is, at the lowest level, achieved by wrapping the double-stranded DNA (dsDNA) around a histone protein octamer into particles known as nucleosomes. These are molecular spools, resembling beads on a string when observed by electron microscopy. In a single nucleosome, a DNA sequence of exactly 147 bp is wrapped around the histone core complex. The DNA needs to be sharply bent so that it can be tightly wrapped around the core proteins. This sharp bending occurs at every helical repeat, which is roughly every 10 bp. Nucleosomes are separated from each other by linker DNA of up to 50 bp. Two recently published, independent studies, one by Segal *et al. *[1] and one by Ioshikhes *et al. *[2], present evidence that the primary DNA sequence can facilitate the bending of the helix around the histone octamer by presenting AA dinucleotides at those positions where the phosphodiester backbone of the helix faces towards the histone core. A sequence favouring nucleosome wrapping is therefore composed of AA dinucleotides spaced approximately 10 bp apart. TT dinucleotides are observed approximately 5 bp in either direction of the AA dinucleotides, as this is the position where the complementary helix faces the core complex. This particular arrangement and periodicity of dinucleotides along a stretch of DNA is referred to as a nucleosome positioning sequence or NPS.

The binding of nucleosomes is highly dependent on the individual DNA sequences. The affinity of different DNA sequences for nucleosomes may vary by 1000-fold or more [1]. The genome thereby appears to encode, at least partly, its own packaging by positioning nucleosomes using these NPSs [3]. Unfortunately, this 'genomic code' is subtle and diffuse and it is often difficult to distinguish it from random noise [2]. This is the main reason why its discovery arrived so many years after the initial deciphering of the regular genetic information stored in DNA. The genomic code for nucleosome positioning could be even more diffuse than previously thought, as the structure of the histone core particle reveals that tetranucleotides or possibly even longer sequences are involved, rather than the dinucleotide patterns that were recently discovered [4]. Despite the diffusely encoded rules, the influence of the DNA sequence has nevertheless been established. However, the picture is somewhat more complex than this. The DNA-nucleosome interaction is dynamic, and the nucleosome occupancy is considered to be in a state of thermodynamic equilibrium. While nucleosomes can bind to almost any DNA sequence, the binding affinity is highly dependent on the specific sequence. Hence, the probability that a certain sequence is occupied by a nucleosome depends partly on the DNA sequence itself, and partly on other factors. These factors include epigenetic profiles and energy-dependent remodeling complexes [5,6], steric hindrance and competition with other DNA binding molecules, such as transcription factors [7], all of which play roles in shaping the actual nucleosome occupancy patterns found along a DNA sequence in vivo.

In this article, we present evidence that nucleosomes could play an important role in the recognition of transcription factor binding sites (TFBS), guiding transcription factors to their target sites on the DNA. The nucleosomes appear to be positioned *in vivo *in such a way that functional binding sites are exposed through a relaxed, open chromatin structure, readily accessible to any transcription factor. On the other hand, the overwhelmingly large number of cryptic (i.e. false positive) binding sites that occur throughout the eukaryotic genome tend to be masked and inaccessibly wrapped in nucleosome particles, which are themselves stacked into a higher-order, dense chromatin configuration. This intriguing hypothesis has been proposed before by others [7], but to the best of our knowledge it has never been fully investigated in higher eukaryotes. Segal *et al. *presented evidence supporting the hypothesis in yeast [1]. In the present paper, we present further evidence based on human data, confirming the hypothesis in higher eukaryotes.

## Methods

To discriminate between true transcription factor binding sites (TFBSs) and false positives, the predicted nucleosome occupancy patterns of the respective surrounding genomic regions were examined.

### Construction of the data sets

Nucleosome positions were predicted for four sets of genomic sequences. The first set includes only sequences that contain a TFBS the existence of which has been experimentally verified, not merely a motif presumed or predicted to be one. This set will be referred to as the 'true positives' set. The second set includes only reference or control sequences, which do not contain, or at least are not very likely to contain, a true TFBS, but that do contain a motif matching that of a true TFBS instead. Hence this set will be referred to as the 'false positives' set. A set of experimentally verified TFBSs was obtained from the *Open Regulatory Annotation Database*, which is also known as ORegAnno [8]. A simple query for human TFBSs whose *in vivo *functionality is supported by experimental evidence resulted in 232 usable true positive TFBSs. Using the genome mappings provided by OregAnno, each TFBS was extracted along with its flanking sequence from the finished human genome assembly (hg18 – March 2006) [9], thus constructing the true positives data set. For this task an extractor script was written in Perl. For each TFBS, 3 kb of flanking sequence was included in both up- and downstream directions. Consequently, all the TFBSs in the data set are aligned: the starting position of the TFBSs is positioned precisely at the 3000th nucleotide in every sequence. The second set, containing false positives, was constructed in a comparable fashion. Using 'findMotif', a tool using an exact string matching algorithm that was written by Jim Kent and included in the UCSC Genome Browser source code [10], we located exact matches of TFBS motifs from the true positives dataset in the human promoters database, 'upstream1000.zip', which contains the coordinates and sequences of the regions 1000 bp upstream to the TSS of human genes and can be downloaded from [11]. The overwhelming majority of these matches are false positive TFBS hits. This assumption is based on the experience that algorithmic TFBS prediction results in an unacceptable high number of hits consisting of a few true functional binding sites and numerous false non-functional binding sites [12]. As demonstrated by Tronche et al. [13] the high number of false predictions is not simply a result of inadequate model frameworks: the predicted sites are effectively bound by transcription factors *in vitro*. In fact the methods do detect potential binding sites, albeit not necessarily functional ones. In a real cell due to the context and the combinatorial nature of regulation only 0.1% of the predicted putative TFBSs will be functional [14]. From the returned matches, five were randomly sampled (unless only five matches or less were returned). Only motifs 5–16 bp long were searched for, as this is the limit imposed by the findMotif program. Next, the absolute chromosomal coordinates (genome mappings) were calculated for each hit. Again, as with the true positives set that was described above, the new set of false positive sequences, containing 3 kb of flanking sequence on each side, was extracted from the hg18 (March 2006) human genome assembly using the genome mappings that were obtained from the wrapper. A total of 694 false positive reference sequences were generated this way. Potential overlap of the false positives data set with the true positives data set was avoided by filtering the false positives data set against the true positives data set and the chip-chip fragment database contained in the Transfac Professional Rel. 11.4 database [15], reducing the number of false positive reference sequences from 694 to 575. Finally, two data sets of 232 random DNA sequences with a length of 6 kb were generated using the random-seq application included in the RSA-tools from van Helden [16]. As both our data sets of experimentally verified TFBSs and false positives are derived from non-coding upstream sequences, we selected this available option in the random-seq tool. One data set contained sequences generated with a 0^th ^order Markov chain model calibrated to obtain sequences with nucleotide frequencies similar to those observed in human non-coding upstream sequences. The other data set contained sequences generated with a 1^st ^order Markov model calibrated to obtain sequences with similar dinucleotide frequencies as human non-coding upstream sequences. This is of importance as dinucleotides are known to be important for nucleosome positioning.

### Nucleosome prediction

The nucleosome arrangement along all of these sequences was predicted using the thermodynamic model published by Segal *et al. *[1], who also provided a 32-bit Linux binary executable, wrapped by Perl scripts and released under the GNU LGPL, especially for this purpose [17]. A regular Linux box running Fedora Core 6 (×86 architecture) was used to carry out the nucleosome prediction calculations. Automation of the entire process was achieved using shell scripts and customized Perl wrapper scripts to process input and output of the binary executable. The binary executable itself was used to calculate the nucleosome occupancy using a nucleosome-DNA interaction model that was developed by Segal *et al. *[1] using yeast sequence data. For each base pair position, P_start _and P_occupied _probabilities are returned by the executable. P_start _is the probability that a particular position is a nucleosome start site. The P_occupied _is the probability that it is occupied by a nucleosome at any point in time. According to Segal *et al. *'stably positioned nucleosomes' are defined by P_start _> 0.2 [1]. Only the stably positioned nucleosomes were extracted from the executable's output, the probabilities at other positions in the sequence were reduced to zero. This is of particular importance, because once several different sequences are examined to find average probabilities at each sequence position, then the less stably positioned nucleosomes will start to constitute noise.

### Evaluation of the nucleosome occupancy

To examine the global nucleosome occupancy distribution in each data set, the nucleosome occupancy probabilities at every sequence position were summed across all sequences in the data set, resulting in the cumulative occupancy probability for every sequence position. Next, the cumulative probability was normalized against the true positives data set. This normalization is necessary to account for the different numbers of sequences in the data sets. Finally, the resulting cumulative probabilities were plotted for each data set using gnuplot [18]. The region surrounding the TFBS could then be examined for a global nucleosome occupancy distribution. The above procedure was repeated iteratively, each time using a different P_start _threshold value for extracting the stable nucleosomes. Finally, for the P_start _threshold value that yielded the biggest difference between the two data sets, a Welch two sample t-test was carried out in R [19] to assess the statistical significance of the observed differences between the true positives data set and the false positives data set. The Welch two sample t-test was used because the populations of P_start _threshold values of the true positives and false positives had unequal variances.

## Results

### Nucleosome occupancies around true and false positive transcription factor binding sites

**Figure 1:**
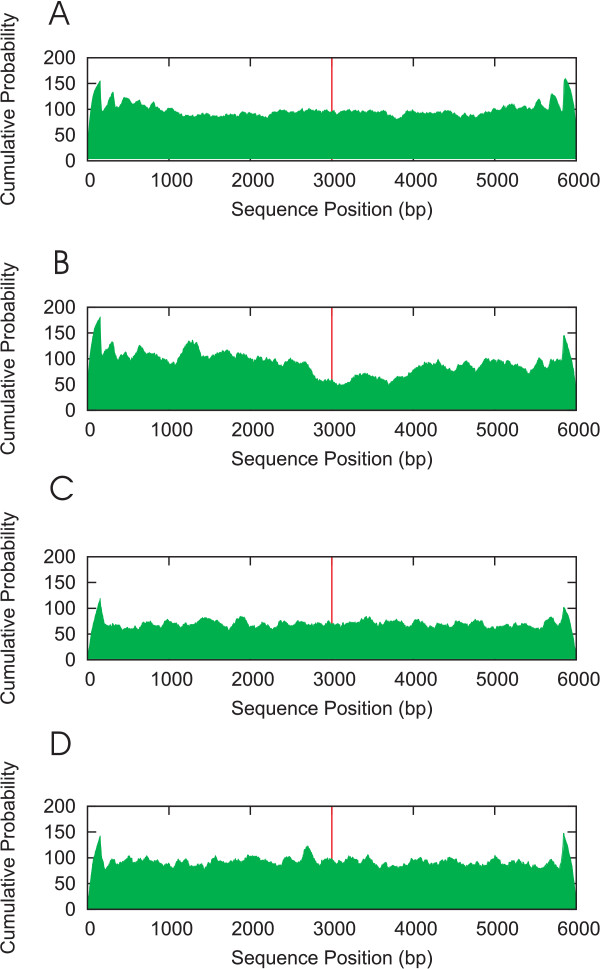
**Figure 1A shows the cumulative probabilities (P_occupied_) for the false positives data set.** P_occupied _is the probability that a position is occupied by a nucleosome at any point in time. The position of the non-functional binding site is indicated by the red line at sequence position 3000. Cumulative nucleosome occupancy is distributed evenly across the complete length of the sequence, indicating a uniform distribution of nucleosome-positioning sequences. Figure 1B shows the cumulative probabilities for the true positives data set. At the position of the functional binding site (red line) a drop in the cumulative nucleosome occupancy is observed relative to the surrounding sequence, indicating that this area is less likely to be occupied by nucleosomes than the other areas located further up- and downstream. Furthermore, the cumulative probability in this area is comparable to the level observed for the random sequences data set: non-genomic sequences generated by a zero-order Markov model (Figure 1C). Figure 1D shows the normalized cumulative probability in the data set of non-genomic sequences generated by a first order Markov model and is distributed evenly along the whole sequence and at the same level as that in the false positives data set. It is also at the same level as that in the true positives data set at a range of positions distant to the true TFBS (for instance the 1–2 kb region upstream of the TFBS).

Figure 1 shows the resulting cumulative probability (P_occupied_) for the four data sets. The P_start _> 0.2 criterion recommended by Segal et al. [1] was used to extract the stable nucleosomes before summing P_occupied_. At the position of the true TFBS (indicated by the red line in the centre of Figure 1B), a drop in the cumulative nucleosome occupancy relative to the surrounding sequence is observed, indicating that the area surrounding the TFBS is less likely to be occupied by nucleosomes than other areas located further up- or downstream. Such a drop is not observed in the false positives data set (Figure 1A), in which the cumulative nucleosome occupancy is distributed evenly along the whole the sequence. The normalized cumulative probability in this data set is at the same level as that in the true positives set at positions distant to a true TFBS (for instance the 1–2 kb region upstream of the TFBS). Likewise, the cumulative probability in the data set of random sequences, generated by a 0th order Markov model, is at a level comparable to that of a region containing a true TFBS (Figure 1C) suggesting the absence of NPSs around true functional human TFBSs. Finally, the normalized cumulative probability in the data set of random sequences generated by a first order Markov model (Figure 1D) is distributed evenly along the whole sequence and at about the same level as that in the false positives data set. It is also at about the same level as that in the true positives data set at positions distant to the true TFBS (for instance the 1–2 kb region upstream of the TFBS).

### Determination of the optimal P_start _threshold value

**Figure 2:**
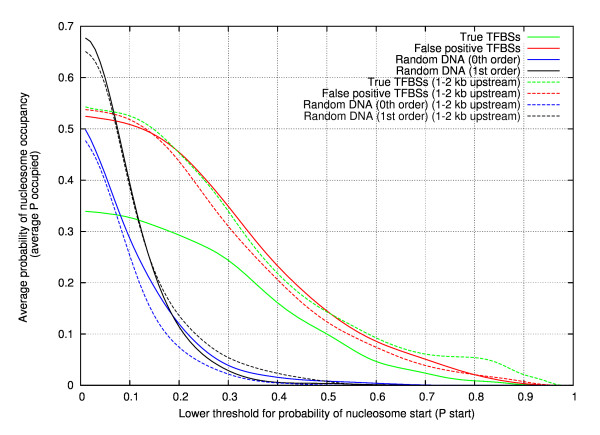
**Determination of the optimal P_start _threshold (α) value.** For every α, ranging from 0.01 to 0.99 in steps of 0.01, the average nucleosome occupancy (P_occupied_) at the true or false positive TFBS site was determined in all four data sets (full lines) and averaged over a range of 1–2 kb upstream (dashed lines). Again, our previous conclusions were confirmed. For low α-values, the average occupancy level of a true TFBS is lower than the average occupancy levels observed with the random sequences (either 0^th ^order or 1^st ^order). Alternatively, the nucleosome occupancy at a false positive TFBS is comparable to the average occupancy observed over a range of 1–2 kb upstream of a true TFBS. For α > 0.1, the nucleosome occupancies for the data sets of random sequences (either 0^th ^order or 1^st ^order), drop quickly reaching very small values. As these values are considerably smaller than the ones obtained with the data set of true TFBSs, this suggests that while low nucleosome occupancy is encoded around true TFBSs, this does not mean nucleosomes should be absent.

**Figure 3:**
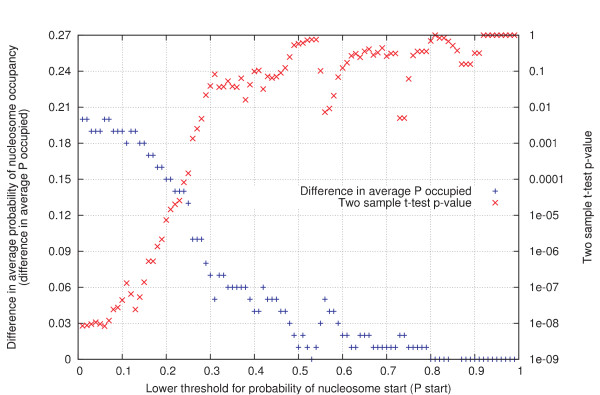
**Statistical evaluation of the difference between the true and false positives data sets.** The difference between the average P_occupied _of the false positives and true positives set is plotted in blue. The maximal difference (0.2) is observed for α = 0.01. For each data point in the graph, a Welch two-sample t-test was performed on the data sets to assess the statistical significance of the difference in their mean P_occupied _values. The obtained p-values were plotted in red. The difference in mean between the true and false positives data sets is most significant for α = 0.01 (p-value = 8.46e-9).

Next, if P_start _threshold = α, the most optimal α to extract the stable nucleosomes was determined. Since the aim is to discriminate as much as possible between the true and false positives data sets, it is essential to establish the threshold value manifesting the largest possible difference between the two sets. Results are presented in Figures 2 and 3. For every α, ranging from 0.01 to 0.99 in steps of 0.01, the average nucleosome occupancy (P_occupied_) at the true or false positive TFBS site was determined in all four data sets (thick, full lines in Figure 2) and averaged, over a range of 1–2 kb upstream (dashed lines). Again, our previous conclusions were confirmed. For low α-values, the average occupancy level of a true TFBS is lower than the average occupancy levels observed with the random sequences (either 0th order or 1st order). Alternatively, the nucleosome occupancy at a false positive TFBS is comparable to the averaged occupancy observed over a range of 1–2 kb upstream of a true TFBS. For α > 0.1, the nucleosome occupancies for the data sets of random sequences (either 0th order or 1st order), drop quickly reaching very small values. As these values are considerably smaller than the ones obtained with the data set of true TFBSs, this suggests that while low nucleosome occupancy is encoded around true TFBSs, this does not mean nucleosomes should be absent.

In addition, the most optimal P_start _threshold value was determined. The difference between the average P_occupied _of the false positives set and true positives set is plotted in blue in Figure 3. The maximal difference (0.2) is observed for α = 0.01. For each data point in the graph, a Welch two sample t-test was performed on the data sets to assess the statistical significance of their difference in mean P_occupied _values. The obtained p-values are in red. The difference in mean between the true and false positive data sets is most significant for α = 0.01, with a p-value as low as 8.46e-9.

### Effect of motif lengths and sampling region on nucleosome occupancy probability

**Figure 4:**
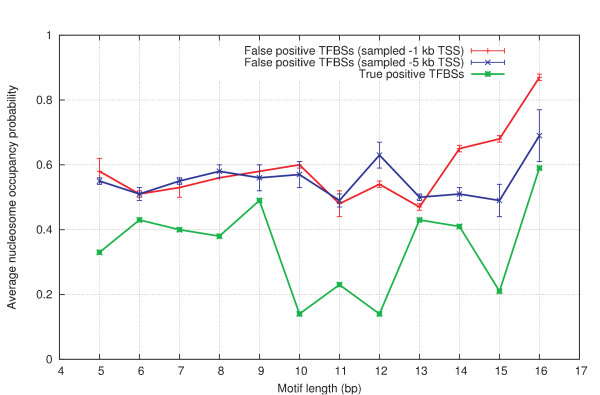
**Effect of motif length and sampling region on false positives data set.** The data set of true positive TFBSs were ordered and grouped by their motif lengths. For each motif, 10 randomly matching hits were searched for in upstream promoter regions (both 1 kb and 5 kb upstream of TSS), and the resulting average nucleosome occupancy (P_occupied_) of these false positives was calculated for each motif length. For comparison, the average nucleosome occupancy was also determined for the true TFBSs grouped per motif length. As can be seen, the reference sets have a higher average nucleosome occupancy for every motif length, indicating that the hits of longer motifs do not enrich the reference data set with true positive hits. Consequently all motif lengths ranging from 5 to 16 bp can be used to construct a reference false positives data set.

**Figure 5:**
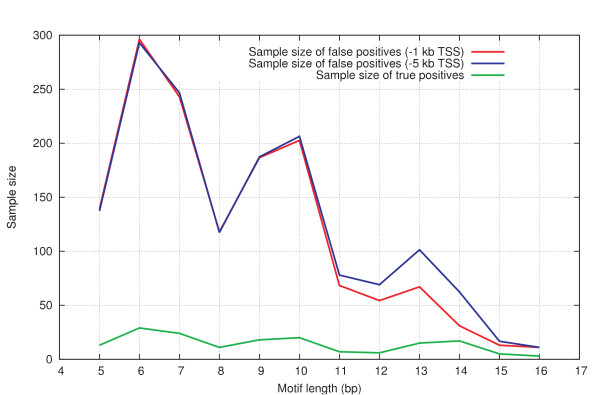
Sample sizes of the different data sets as a function of their constituting motif lengths.

The effect of motif length on the false positives data set was checked, because a search for longer motifs (e.g. >10 bp) might have a significantly increased chance of returning true positive hits in addition to the false positive hits. The original 232 true positive TFBSs were ordered and grouped by their motif lengths. For each motif, 10 randomly matching hits were searched for in upstream promoter regions (both 1 kb and 5 kb upstream of TSS), and the resulting average nucleosome occupancy (P_occupied_) of these false positives was calculated for each motif length. For comparison, the average nucleosome occupancy was also determined for the true TFBSs grouped per motif length. Results can be found in Figure 4 and sample sizes are plotted in Figure 5. The reference sets have higher average nucleosome occupancy for every motif length. The reference sets also have a very stable average nucleosome occupancy that does not start to decline towards the longer motif lengths even if the difference with the true TFBSs is considered. It appears that the hits of longer motifs do not enrich the reference data set with true positive hits, and consequently all motif lengths ranging from 5 to 16 bp can be used to construct a reference false positives data set. This is important because these longer motifs are contained in the true positives data set, and so in order to make a fair comparison, an equal fraction should preferably also be represented in the false positives set.

There is little difference between the average nucleosome occupancy probabilities of the two reference sets. Each set was sampled from a region spanning a different length upstream of the TSS. The reference set sampled up to 1 kb upstream of the TSS and has a lower standard deviation for most data points, indicating that in a smaller region there is less choice in motif matches to randomly sample from. This is supported by the difference in sample sizes for the longer motif lengths. However, the larger standard deviation of the reference set that was sampled up to 5 kb upstream of the TSS can also be ascribed to the possibility that this reference set exceeds the boundaries of the promoter region, and as such it could also contain regions with a different bias in nucleosome occupancy. Either way, although the average occupancies between the two data sets do not differ greatly, sampling from a region up to 1 kb upstream of the TSS is preferential because it yields a more 'stable' false positives data set.

## Discussion

The computational prediction of TFBSs from a nucleotide sequence alone results in an unacceptable high number of false positives in the predicted sites. This has been termed the futility theorem by Wasserman et al. [14] as nearly every predicted TFBS has no function *in vivo*. To improve the accuracy of the predictions, new approaches take into account information in and beyond the TFBSs, such as the preferences of binding site locations and the co-occurrences of other motifs in promoter regions [20,21].

In the present study, we evaluate whether nucleosome occupancy around functional human TFBSs is a powerful enough feature to include in TFBS prediction algorithms as otherwise its inclusion would result in the addition of extra noise to the binding site detection system without the yield of more prediction accuracy. For that purpose, the nucleosome occupancy (density) around true TFBSs was compared to that around cryptic or false positive TFBSs using large human data sets and the significance of the difference between the two data sets was then statistically evaluated.

Comparison of true positives data sets with false positives data sets should be a reliable, genuine and fair. Therefore it is of particular importance to construct a correct reference (false positives) data set. Thus, when constructing a reference data set by collecting reference sequences, care has to be taken not to introduce a bias in the nucleosome occupancy. The human genome contains many regions that could easily result in such a bias: for instance, intergenic, intronic, exonic, and promoter regions probably have different nucleosome occupancy distributions due to differences in frequency and in organization of NPSs in each of these regions. Ultimately, it would be impossible to tell whether a significant difference from the reference data set with the true positives set was due to the true TFBS itself, to the bias of the region it is contained in, or to a bias in the reference set. To eliminate this uncertainty, we ensured from the beginning that the reference set and true positives set showed the same bias. Since most TFBSs are located in gene promoters, only promoter regions were used in the reference set. These promoter regions are located immediately upstream of the transcription start sites (TSS) of genes. How far upstream of the TSS they actually start is vague and differs from gene to gene. A reference set containing promoter regions could, at best, contain the first × kb upstream of the TSSs of annotated genes, where × is a safe number. Traditionally, a region up to 1.5 kb upstream of the TSS is scanned for putative TFBSs, with most TFBSs located within the first 0.5 kb. For that reason, a region spanning the first 1 kb upstream of the TSS was used to construct a false positives data set. Unfortunately, there are no databases for false TFBSs. To construct a false positives data set, random hits of each TFBS motif from the true positives data set were located in promoter regions, and using the returned genome coordinates, the flanking sequences were excised from the human genome assembly. This approach relies on the fact that by chance alone a short sequence motif, such as that of a TFBS, comprising only a few base pairs in length, occurs in a random genomic region at a very high frequency. This is caused by the limitation of the nucleic acids alphabet to only four bases. Typically, most of these hits are not true TFBSs but cryptic ones, i.e. the motifs occurs by chance. Randomly sequences generated *in silico *were also used as a reference. A randomly generated sequence, i.e. a non genomic sequence, is not likely to contain the recurring dinucleotide patterns, every 10 bp or so, that are required for a tight nucleosome-DNA interaction. It is therefore useful to establish the basal nucleosome occupancy in the biological system, or at least the basal level predicted by the thermodynamic model. In a genomic sequence, a lower nucleosome occupancy level will not be predicted.

Our results (Figure 1) suggest that the genomic sequence immediately surrounding a true TFBS does not contain any 'instructions' in the form of patterns for binding nucleosomes – just like *in silico *generated noise does not and cannot contain these either. Note, however, that this does not mean that these regions should be free of nucleosomes *in vivo*. The nucleosome-DNA interaction is in thermodynamic equilibrium. Several factors affect the binding of the nucleosomes: steric effects, competing molecules (such as transcription factors), nucleosome remodeling complexes, and genomic instructions. The genomic instructions merely help to push the equilibrium in the direction of higher nucleosome occupancy. In the absence of these genomic instructions, nucleosomes will still be able to bind to the DNA, but with reduced affinity, and hence with both a reduced likelihood to occupy the particular region and an increased susceptibility towards shifting positions. Considering the results depicted in Figures 1, the genomic instructions seem to be remarkably absent around true TFBSs, making them more accessible to transcription factors.

As discussed above, the region where false positive hits are sampled is of particular importance. The observed drop in nucleosome occupancy where a true TFBS is positioned could be, for instance, due to generally reduced nucleosome occupancy specific to the region proximal to the TSS. The first 1 kb upstream of the TSS most often contains several cis-regulatory elements (CREs) and would thus benefit from a generally open chromatin structure. As a result, the observed drop in nucleosome occupancy would not be specific for the particular (functional) TFBS anymore, but for the whole region. If false positive hits were to be sampled from a very large promoter region (e.g. up to 5 kb upstream of TSS), the majority of the hits would not originate from the first 1 kb, where most true TFBSs are positioned, but from regions more upstream, where the chromatin structure is likely to be more closed. Thus, false positive hits must be sampled from the region in which the functional TFBSs are located. If true TFBSs were enjoying an open chromatin structure specific merely to the region they are located in, then the false positive hits would also exhibit lower nucleosome occupancy. This was not the case, as demonstrated in Figure 4, indicating that the lower nucleosome occupancy was specific for the true TFBS and not for the region it was contained in.

## Conclusion

We present evidence in higher eukaryotes supporting the hypothesis that transcription factors are guided to their target sites on the DNA by the nucleosome configuration. The positioning of these nucleosomes is intrinsically encoded by the genome itself, through recurring dinucleotide patterns that interact with the histone core of a nucleosome particle. Investigation of these nucleosome positioning sequences (NPSs) revealed that they were remarkably absent around functional TFBSs compared to the reference sequences (false-positive TFBSs). The absence of these patterns around true TFBSs does not necessarily mean the absence of nucleosomes. A reduced probability that the particular sites will be occupied by nucleosomes can be expected, because the absence of these positioning patterns will weaken the nucleosome-DNA interaction, but it will not prevent it. As a result, functional TFBSs are less likely to be blocked by dense chromatin configurations. Put differently, they are more likely to be exposed and thus accessible to their transcription factors. This is of particular importance, as it allows the *in silico *discrimination between functional TFBSs and the far more numerous false positive TFBSs that are found in the human genome. It could thereby substantially improve current TFBS prediction techniques, which are based on sequence conservation and tend to yield too many false-positive hits.

## Authors' contributions

FD and PJDB did the bioinformatics analysis, FvR, PJDB and FD contributed to the design of the study, PJDB designed and coordinated the study. All the authors contributed to the writing of the paper. All the authors read and approved the final manuscript
